# Spatial Dynamics and Sterilization Range of Incompatible *Aedes albopictus* Males: Advancing Toward an Optimized IIT Approach

**DOI:** 10.3390/tropicalmed11020045

**Published:** 2026-02-06

**Authors:** Elena Lampazzi, Chiara Virgillito, Beniamino Caputo, Giulia Lombardi, Greta Santarelli, Riccardo Moretti, Maurizio Calvitti

**Affiliations:** 1 Italian National Agency for New Technologies, Energy and Sustainable Economic Development (ENEA), 00123 Rome, Italy; elena.lampazzi@enea.it (E.L.); giulia.lombardi.1993@gmail.com (G.L.); 2 Department of Public Health and Infectious Diseases, Sapienza Università di Roma, 00185 Roma, Italy; chiara.virgillito@uniroma1.it (C.V.); beniamino.caputo@uniroma1.it (B.C.); greta.santarelli@uniroma1.it (G.S.)

**Keywords:** *Wolbachia*, *Aedes albopictus*, population suppression, open-field trials, incompatible male survival, incompatible male dispersal, egg sterility, incompatible insect technique (IIT), generalized linear model (GLM), genetic marker

## Abstract

The Incompatible Insect Technique (IIT) is a species-specific, eco-friendly mosquito control method that relies on releasing *Wolbachia*-infected males, which induce cytoplasmic incompatibility (CI), rendering eggs inviable when mating with wild females. Aiming at optimizing IIT protocols in terms of cost-effectiveness, data on incompatible male dispersal and survival and the distance- and time-related impact of induced sterility are fundamental. This study plans to fill this gap and reports findings from a two-year field trial (2022–2023) at the ENEA-Casaccia Research Center, based on single-spot releases of incompatible *Aedes albopictus* males (AR*w*P strain). Male releases were carried out in late September 2022 (~15,000 released males) and the early *Ae. albopictus* season (at the end of June 2023; ~24,000 released males). Fifty-eight ovitraps were located at a 20–900 m distance from the AR*w*P release spot and were monitored weekly from May to November to assess egg hatching rates and measure CI effects in relation to both distance and time. Following the 2023 release, samples of adults were collected at increasing distances from the release site and at multiple post-release time points to assess, individually, wild female fertility and AR*w*P male dispersal and survival using *Wolbachia* as a genetic marker. Statistical analyses revealed that: (a) the highest reduction in the egg hatching was found within 100 m from the release spot (46.5% and 19.9%, respectively, in 2022 and 2023) but remained significant even at greater distances (29.9% and 7.7% at 300 m, respectively, in 2022 and 2023); (b) accordingly, the highest reduction in the wild female fertility occurred within 100 m from the release spot (47.3%), but similar effects were recognizable up to 600 m; (c) the overflooding ratio of the AR*w*P males did not significantly differ between 3 and 11 days after the release, with AR*w*P males remaining active up to 18 days and dispersing as far as 400 m. These results demonstrate the potential of localized, non-inundative IIT trials to furnish clues for the setup of spatially optimized release strategies, especially in scaled-up applications. The study also emphasizes the need for standardized assessment tools and further research regarding environmental and behavioral factors influencing long-term suppression outcomes.

## 1. Introduction

In recent decades, Europe, particularly the Mediterranean region, has witnessed a marked expansion in mosquito species that act as vectors of human and animal pathogens. Notably, invasive species such as *Aedes albopictus* and, more recently, *Aedes aegypti* have established themselves in new territories, while native species like *Culex pipiens* are exhibiting an increasing impact on the epidemiology of the viruses they transmit [1,2].

This emerging scenario is driven by multiple interrelated factors, including climate change, which has led to rising average temperatures and altered precipitation patterns, heightened global mobility and trade, and the intrinsic ecological plasticity of these mosquito species, enabling rapid adaptation to urban and peri-urban environments [3,4]. Consequently, the risk of transmission of pathogens such as dengue, chikungunya, Zika, yellow fever and West Nile viruses—historically considered distant threats to Europe—is steadily increasing.

Compounding this challenge is the widespread development of insecticide resistance, resulting from the prolonged and intensive use of conventional chemical control methods [5]. This phenomenon diminishes the efficacy of traditional vector management strategies, complicates containment efforts, and facilitates the persistence and spread of invasive mosquito populations.

As a result, current mosquito control programs in Europe face increasing challenges in terms of effectiveness, sustainability, and public acceptance, highlighting the urgent need for innovative, environmentally sustainable, and community-oriented interventions that can complement or replace conventional control methods in complex urban environments. Effective approaches should integrate advanced surveillance systems, targeted ecological interventions, and safe biotechnological tools, in line with the principles of the One Health framework, which recognizes the interconnection between human, animal, and environmental health.

Among the array of vector control strategies explored in recent decades, genetic control approaches have emerged as particularly promising [6]. These methods leverage species-specific biological mechanisms to suppress or modify mosquito populations to decrease their vectorial capacity, offering a high degree of precision while minimizing non-target impacts. The classical Sterile Insect Technique (SIT) is a cornerstone of this category. SIT involves the mass rearing and systematic release of male insects sterilized through exposure to ionizing radiation. The released males compete with wild males for mating opportunities, leading to infertile matings and, over time, to a progressive reduction in the reproductive capacity of the target population [7,8,9]. Building on SIT, a derivative strategy known as the Incompatible Insect Technique (IIT) represents an innovative control approach aimed at suppressing mosquito vector populations and relies on the large-scale release of laboratory-reared male mosquitoes that are reproductively incompatible with wild females [10,11,12,13]. Incompatibility is caused by the infection of specific strains of the endosymbiotic bacterium *Wolbachia* [14] and consists of a form of embryo lethality resulting from matings between infected males and uninfected females or females infected with a mismatched strain (cytoplasmic incompatibility = CI) [10,12]. As *Wolbachia* is maternally transmitted, the development of transinfection techniques has enabled the establishment of mosquito lines carrying selected strains of the bacterium that confer complete CI, allowing the use of males as an effective sterilizing tool without the need for irradiation or other pre-release treatments [10,12,15].

IIT-based suppression programs are run in several areas of the world [10], but IIT already showed promising potential in Europe too when open-field trials against wild *Ae. albopictus* populations led to a significant reduction in egg hatching. This was despite a low average ratio between incompatible and wild males (close to 1:1) and even though the treated areas were not insulated from incompatible male dispersal and indigenous female immigration [16,17]. These recent Italian pilot trials suggest that even a limited quantity of incompatible males may effectively reduce the mean fertility of wild urban populations, provided that male mating competitiveness and life expectancy are preserved and remain comparable to those of wild males.

Despite their demonstrated potential, both SIT and IIT are operationally demanding and resource-intensive approaches [10,18]. Furthermore, their effectiveness strongly depends on the sterile male mating competitiveness, on the ratio between sterile and wild males, on the spatial configuration and frequency of the releases, and on the duration of the release programs [8,10,19]. All of these parameters are often defined empirically and with limited field-based evidence, particularly in heterogeneous urban landscapes.

As regards IIT against *Ae. albopictus*, there is a critical lack of field-derived, spatially explicit data quantifying how far released incompatible males disperse, how long they remain active, and how the induced CI effect decays with distance and time from a release spot. This information gap limits the ability to design spatially and temporally optimized, cost-effective, and scalable IIT-based suppression programs.

With the aim of responding to this need, this manuscript presents field data from a two-year pilot trial (2022–2023) involving non-inundative releases of incompatible *Ae. albopictus* males conducted at the ENEA-Casaccia Research Center (Rome, Italy). The primary objective of the described experiments was the study of the spatial and temporal dynamics of CI-induced sterility under field conditions by assessing distance- and time-dependent changes in egg hatch rates and wild female fertility. The dispersal and persistence of released incompatible males were also assessed using genetic markers. By addressing these key operational knowledge gaps, this work aims to provide evidence-based guidance to improve the efficiency, economic sustainability, and scalability of IIT-based *Ae. albopictus* control.

## 2. Materials and Methods

### 2.1. Incompatible Male Production and Sexing

Incompatible males belonged to a laboratory colony developed by ENEA in 2008 through the replacement of the natural *Wolbachia* infection of *Ae. albopictus* with a *Wolbachia* strain caught from *Cx. pipiens molestus* [20]. Over the following years, this line, named AR*w*P, was carefully characterized to ascertain the stability of the infection, the level of induced CI, its suitability for mass rearing settings, and its capability to reduce the mean egg fertility of wild *Ae. albopictus* populations both under semi-field and open-field conditions [16,17,21,22,23].

AR*w*P males used during the present trials were produced at ENEA-Casaccia laboratories. Rearing was carried out at a larval density of 2 larvae mL^−1^ in deionized water, at 28 °C, with 80% relative humidity (RH), and at a 14 h:10 h light:dark photoperiod. Larvae were fed for 4 days with increasing doses (0.2, 0.4, 0.6 and 0.8 mg larva^−1^ day^−1^) of a liquid diet consisting of 50% tuna meal, 36% bovine liver powder, and 14% brewer’s yeast (similar to the IAEA-BY diet [24]). Twenty-four-hour-old pupae were sexed mechanically by passing them through a 1400-μm metal sieve over 3 min at 34 °C [25]. After emergence, adults were kept at 15 °C for 24 h and then knocked down by chilling to 10 °C, allowing for manual removal of any residual females. Selected males were kept at 25 ± 1 °C and 80% RH in cubic plastic cages (30 cm; Bugdorm1, MegaView Science Co., Ltd., Taichung, Taiwan) and fed with 10% sugar solution until release.

### 2.2. Study Area: Monitoring of the Ae. albopictus Population and Deployment of the Incompatible Males

**Figure 1:**
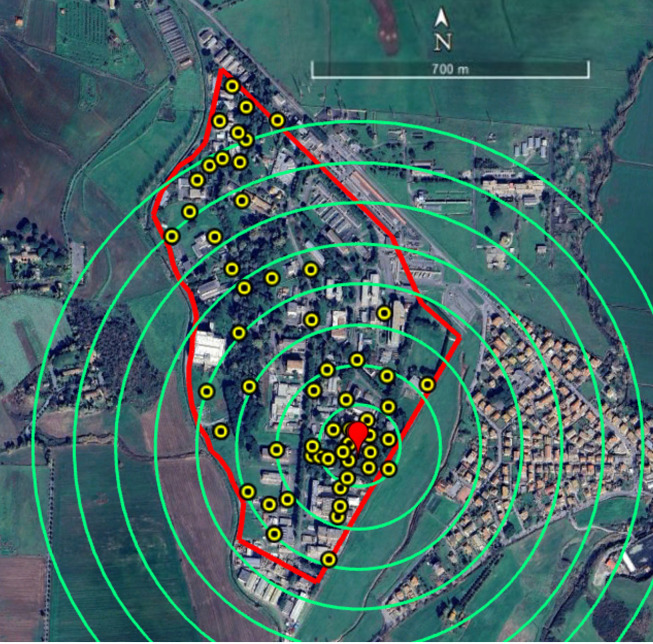
Study area at ENEA-Casaccia Research Center (Rome, Italy), where experiments were conducted. The border of the Research Center is represented in red. Yellow dots identify the positions of the ovitraps (*n* = 58). The red pointerindicates the release spot of *Ae. albopictus* AR*w*P males. Green circles define the distance ranges (100 m each) from the release spot used to analyze the data from ovitraps and HLC.

The study was conducted at the ENEA-Casaccia Research Center, located in a suburban area north of Rome (Coordinates (UTM, WGS84, Zone 33T): Easting: 276,960 m, Northing: 4,656,900 m). The site’s relative isolation from other humanized areas and the possibility of producing incompatible males on site made this location ideal for this type of study. The study area spans approximately 60 ha, with a maximum length of 1.2 km and a maximum width of 600 m, and comprises office buildings, wooded and open green spaces, and paved roads. On average, around one thousand employees work on site, with peak human activity occurring primarily between 9:00 a.m. and 4:00 p.m. In addition to the wild fauna, various colonies of domestic cats are uniformly distributed across the campus. From an aerial perspective, the area presents an irregular shape that can be approximated by an isosceles triangle, with the base fence (approximately 1.2 km) oriented toward the west, and the two side fences (each approximately 700 m) extending toward the northeast and southeast. The area where males were released (the release spot) consisted of a rectangular plot measuring 50 m in length and 10 m in width (Figure 1). Its location was selected to enable a study range of no less than approximately 700 m within the Research Center.

The population dynamics of *Ae. albopictus* in the study area was investigated over two years (2022–2023) with ovitraps, starting on May 1st and ending in the first week of November. For this purpose, 58 ovitraps were placed in shaded locations grouped into 100 m-wide concentric circular bands radiating from the release spot, extending up to 700 m, as shown in Figure 1. Due to the shape of the study area, not all circular bands were complete. Ovitraps consisted of black plastic vases with an overflow hole 3 cm below the upper border, filled with 600 mL of tap water. A wooden paddle with one rough side was placed in each ovitrap. Each ovitrap was assigned a dual identification code consisting of a number, related to a specific circular band around the release spot, and a letter to identify different traps in the same band. Additional traps with an “M” prefix (e.g., M1, M10) were placed at more distant, randomly selected locations, in an area outside the concentric arrangement. Each ovitrap was clearly labeled on-site according to its alphanumeric code, allowing unambiguous identification throughout the study (Table S1).

The paddles were replaced weekly and were then kept in the laboratory under controlled conditions (26 ± 1 °C; 60 ± 10% relative humidity) for 5 days to allow for complete embryo development. After this period, the paddles were submerged in rainwater for 24 h to allow the larvae to hatch. Egg hatching rates were then calculated based on the proportion of hatched eggs out of the total number of eggs that were examined.

In 2022, three single-spot releases of 5000 AR*w*P males were conducted weekly in September, beginning on the 8th. In 2023, releases of 9000 and 15,000 AR*w*P males occurred, respectively, on 22 and 28 June. All releases were performed in the late afternoon between 5:00 and 7:00 p.m. to promote male adaptation to the new environment while avoiding exposure to high temperatures. Throughout the duration of the experiments, climatic data on temperature, humidity, rainfall, and wind parameters were recorded using a Davis Vantage Pro 2 weather station (Davis Instruments, Hayward, CA, USA) working in the Research Center.

### 2.3. Distance-Related Ae. albopictus Egg Hatching Rate Following Ae. albopictus ARwP Male Releases

A Generalized Linear Model (GLM) with a binomial distribution with logit link function was implemented to evaluate the effects of ARwP male releases on the hatching rate of eggs collected by ovitraps. The hatching rate, calculated per ovitrap and sampling week, was analyzed relative to the distance from the release spot through a pre- and post-treatment comparison. The pre-treatment phase included eggs collected during the two weeks prior to release, while the post-treatment phase comprised eggs collected during the two weeks following the releases (Figures S1 and S2). In 2022, the pre-treatment phase covered the period from 25 August to 7 September, while the post-treatment phase covered the period from 29 September to 12 October. In 2023, the pre-treatment phase refers to the period 9–22 June, and the post-treatment phase to the period 30 June–13 July.

The GLM included pre/post as a qualitative variable, the distance from the release spot as a quantitative variable, and their interaction as covariates to model the temporal effect of hatching across the pre- and post-treatment phases. Separate GLMs were implemented for each year. In the model for the AR*w*P releases carried out in 2022 (GLM-1), the three releases were treated as a single event (for a total number of 15,000 released males). Similarly, the model for the AR*w*P releases carried out in 2023 (GLM-2) considered releases as a single event (for a total number of 24,000 released males).

### 2.4. Field Collection of Ae. albopictus Females and Evaluation of Their Fertility in Relation to Distance from the Male Release Spot

During the 2023 experimental season, *Ae. albopictus* females were collected in the study area on June 16th (before releases) and 2, 7, and 14 days after the 28 June release to investigate their fertility in response to the incompatible male releases (Figure S3). Operators (the authors E.L., G.L., and M.C.) remained stationary for five minutes in late afternoon (between 5:00 and 7:00 p.m.) at random locations included within circular 100 m-wide bands at increasing distance from the release spot and collected the females by human landing catch (HLC). As soon as a female landed on the operator’s skin, it was captured using a manual aspirator before it could sting. This procedure was approved by the ENEA Bioethical Committee, also based on the absence of arbovirus circulation in the area. For each sampling date, a maximum limit of 65 collected females was established, evenly distributed across the different distance bands. This limit was set for practical reasons. Following capture, individual females were placed in Falcon tubes and transported to the ENEA laboratories, where they were blood-fed on the following day. On Day 3 (D3) post-blood-feeding, each female was transferred to an oviposition cup lined with filter paper and allowed to lay eggs over the next four days. Eggs were processed according to the same protocol used for those collected via ovitraps and were subsequently classified as hatched or unhatched to measure egg fertility. Adult females were then frozen and stored at −20 °C. The spermathecae of the females that only produced non-viable eggs were examined under a microscope to assess insemination.

The hatching rates of single *Ae. albopictus* wild females per sampling week were assessed using a statistical GLM. A GLM-3 was implemented based on the principles of a pre- and post- treatment analysis previously described. A binomial distribution was used for the response variable, and pre/post, distance from the release spot, and their interaction were included as covariates. The pre-treatment phase corresponded to single *Ae. albopictus* females collected on 16 June (when incompatible male releases had not yet occurred), while the post-treatment phase corresponded to those collected on the 1st, 6th, and 12th of July.

### 2.5. Field Collection of Ae. albopictus Males to Measure ARwP Male Survival and Dispersal Capacity

Following the 2023 release, samples of males were collected at increasing distances from the release spot (ranging from 0 to 500 m, at 100 m intervals) and at multiple time points post-release (3, 11, 18, and 22 days). Collections were conducted in the late afternoon (between 5:00 and 7:00 p.m.) using both entomological nets and modified microaspirators (2.5 m/s). Captured males were preserved in tubes containing 70% ethanol for subsequent laboratory analysis. To distinguish between incompatible (AR*w*P) and wild males, molecular identification techniques were employed. Specifically, a PCR assay was used to detect the presence of the *w*Pip strain of *Wolbachia*, which is unique to AR*w*P males. This genetic marker enables accurate identification of incompatible males within field-collected samples and allows for the calculation of their frequency relative to the total male population. These data were used to evaluate the dispersal and the survival of the incompatible males over the days following the release.

Ratios of AR*w*P to wild *Ae. albopictus* males were calculated following the 28th of June release. To assess whether the AR*w*P:wild male ratio differed significantly over time, χ^2^ tests were performed to compare values obtained at 3, 11, 18, and 22 days post-release within each distance interval.

All statistical analyses were carried out using R software v4.4.1.

### 2.6. Molecular Identification of wPip Wolbachia in Field-Collected Specimens

DNA was extracted by homogenizing the abdomens of male mosquitoes in 100 μL STE with 0.4 mg/mL proteinase K. In total, 27 AR*w*P individuals were identified by polymerase chain reaction (PCR) using primers *w*PF (5′-CGACGTTAGTGGTG-CAACATTTA-3′) and *w*PR (5′-AATAACGAGCACCAGCAAAGAGT-3′), which amplify a DNA sequence specific to the *Wolbachia w*Pip strain [13]. The PCR conditions used were 94 °C for 5 min followed by 32 cycles at 94 °C for 30 s, at 54 °C for 30 s, at 72 °C for 40 s and a single final step at 72 °C for 10 min. Amplified fragments were electrophoresed on 1.5% agarose gels, stained with ethidium bromide (1 μg mL^−1^), and visualized under UV light.

## 3. Results

### 3.1. Ae. albopictus Egg Hatching as a Function of Distance from the Release Spot

**Figure 2:**
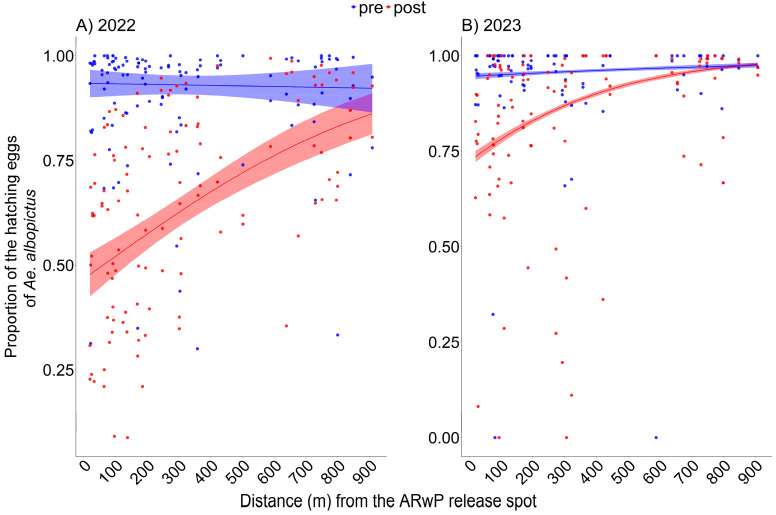
Mean proportion of the hatching *Ae. albopictus* eggs out of the eggs collected by ovitraps as a function of distance (m) from the release spot following incompatible AR*w*P male releases in 2022 ((**A**); GLM-1) and 2023 ((**B**); GLM-2). Data regarding the pre- and post-treatment phases are reported, respectively, in blue and red. Dots = observed (trap-specific) proportion of hatching eggs before (blue) and after (red) the AR*w*P releases. (**A**): 2022; pre-treatment phase = 25 August–7 September, post-treatment phase = 29 September–12 October; (**B**): 2023; pre-treatment phase = 9–22 June, post-treatment phase = 30 June–13 July. Dashed area = 95% confidence interval.

In 2022, a curvilinear statistically significant relationship between egg hatching rate and distance from the release spot was found (Figure 2A, Table S2; GLM-1), with no evidence of systematic patterns in the residuals (Figure S4A,B). The reduction in the hatching rate between pre- and post-treatment phases was higher within 0–100 m from the release spot (46.5%; 95% CI: 45.3, 47.6) compared to the other distance intervals (e.g., 29.9% at 300–400 m). Difference decreased as a function of distance but remained significant across the entire study area (Table S3).

A similar curvilinear statistically significant relationship was also found in 2023 (Figure 2B, Table S2; GLM-2), with no evidence of systematic patterns in the residuals (Figure S4C,D). The reduction in the egg hatching rate between pre- and post-treatment phases was higher within 0–100 m from the release spot (19.9%; 95% CI: 18.8–21%) compared the other distance intervals (e.g., 7.7% at 300–400 m) and decreased as a function of distance until it became not significant above 750 m (Table S3).

### 3.2. Wild Ae. albopictus Female Fertility as a Function of Distance from the Release Spot

**Figure 3:**
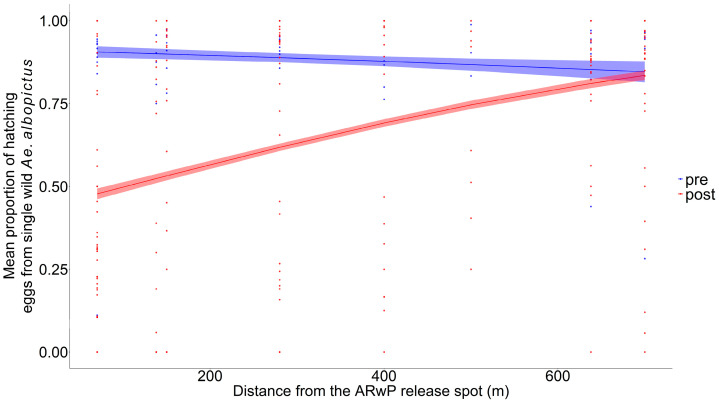
Mean proportion of the hatching eggs produced individually by *Ae. albopictus* females collected in the study area before incompatible male releases (pre-treatment phase, in blue) and after incompatible male releases (post-treatment phase, in red) as a function of distance from the release spot (GLM-3) at ENEA-Casaccia Research Center (Roma, Italy); dots = observed proportion of fertile eggs from each single female (blue dots: pre-treatment phase; red dots = post-treatment phase). Pre-treatment phase collection = 16 June; AR*w*P males releases: the 22 and 28 June 2023; post-treatment phase collections = the 1, 6 and 12 July. Dashed area = 95% confidence interval.

Specifically, 2166 eggs from 65 females were analyzed as representatives of the pre-treatment phase, while 63 (=2699 eggs), 62 (=2780 eggs), and 65 (=2722 eggs) females were collected, respectively, 2, 7, and 14 days after the last incompatible male release to highlight the effects of the treatment (Table S4). The GLM-3 showed a statistically significant reduction in wild female fertility comparing pre- and post-treatment phases up to 600 m (Figure 3, Table S5), with no evidence of systematic patterns in the residuals (Figure S4E,F). The higher reduction between the pre- and post-treatment phases (47.3%; 95% CI: 44.9–49.6%) was found within 0–100 m from the release spot and then decreased with distance (30% at 300 m) (Table S6).

### 3.3. Spatio-Temporal Dynamics of the Ratio Between Incompatible and Wild Males

The average AR*w*P:wild male ratio within the first interval of distance and at 3 days from the last release was 1.58. A not significantly different ratio was observed after another 8 days (χ^2^ = 0.03, *p* < 0.05; 3 days = 1.58, 11 days = 1.73), while a significant decrease in the presence of AR*w*P males was observed 18 days after the release (χ^2^ = 55.4, *p* < 0.05), when the last incompatible males were recaptured (Table 1). Overall, the observed mean proportion of the AR*w*P males decreased significantly with distance from the release spot (*t*-test, *p* = 0.0475). This trend does not seem to be confirmed by the data related to 18 days after the release, but this phenomenon could be due to later migrations of the individuals. Notably, the farthest distance at which AR*w*P males were recaptured was at 300–400 m from the release spot and after 11 days (Table 1).

## 4. Discussion

The primary objective of this study was to evaluate, through an experimental approach involving localized, low-density releases of incompatible *Ae. albopictus* AR*w*P males, the spatial extent of *Wolbachia*-induced CI effects, as measured by the egg fertility variation at increasing distances from a release spot.

Additionally, we aimed to assess the dispersal capacity and the field longevity of AR***w***P males by capturing individuals within the experimental area and the fertility of single wild females collected within a range of 700 m from the release spot. Over the two-year study period, we concurrently monitored vector oviposition activity and hatching rates from May through November.

Results obtained from the ovitraps were quite similar across the two years, despite the strongest reduction in the egg fertility being obtained in 2022. However, considering the overall classes of distance *(*GLM-4) up to 900 m, the egg hatching reached the pre-treatment values more rapidly in 2022 compared to 2023 (Table S7). The steeper spatial gradient observed in 2022 suggests a faster decline of the sterilizing effect with distance, whereas in 2023, the effect appeared more spatially homogeneous. This pattern may reflect differences between the population dynamics of *Ae. albopictus* in the study area that could have been at least partly associated with climatic conditions (Figure S5) and seasonality.

In interpreting the results, it should also be considered that, during the autumn season, the proportion of eggs entering diapause [26] gradually increases, and diapausing eggs may be mistakenly classified as unfertile eggs. This could lead to an overestimation of the CI effect, particularly in October 2022. The pre- and post-treatment experimental design may have further influenced the observed hatching rate patterns, with larger pre–post differences recorded in 2022 (late season) and smaller differences in 2023 (early season) (Figure S6). These sources of bias could be mitigated by estimating fertility using egg viability rather than hatching rates (i.e., dissecting all unhatched eggs to verify the presence of embryos) and by adopting a control site design to complement the pre- and post- treatment comparison.

In accordance with the data from the ovitraps, the study of single females collected at varying distances from the AR*w*P male release spot revealed that those experiencing the highest levels of induced sterility were predominantly located near the release area. However, the CI effects were still detectable up to 500 m. Beyond this distance, female fertility returned to baseline levels, consistent with populations unexposed to sterile males. Previous studies similarly demonstrated that the flight range of most of the *Ae. albopictus* females in search of oviposition sites does not exceed 200 m in peri-urban environments [27], but higher distances can be reached in a few days [28,29]. However, almost all individuals generally remain within 1 km of the release spot [30].

Studying the dispersal capacity of the AR*w*P males is essential to fully understand how egg sterility is induced as a function of distance from the release spot. The possibility of using *Wolbachia* as a genetic marker allowed for the easy distinction of AR*w*P from wild individuals without the need for any preliminary treatment that could result in a bias [17,31,32]. Differently, the use of fluorescent powders for marking *Ae. albopictus* in Mark-Release-Recapture (MRR) and SIT studies has been shown to introduce methodological biases that may affect survival, dispersal, and behavioral parameters [32,33,34,35]. Furthermore, fluorescent powders may be progressively lost due to abrasion, grooming behavior, rainfall, or contact with vegetation, leading to false negatives during recapture, especially in long-term or low-recapture studies [34], and fluorescent powder particles may also be transferred from marked to unmarked mosquitoes through physical contact, aggregation at resting sites, or mating, leading instead to false positives [36]. These issues are of particular concern in SIT programs, where accurate assessment of male sexual performance is essential for evaluating intervention efficacy [37].

The use of *Wolbachia* as a marker has already been demonstrated as neutral to *Ae. albopictus* fitness in comparison with the use of dusts [32]. In the present study, this approach allowed us to highlight that while most of the incompatible males remained clustered near the release spot, some of them were capable of dispersing at least up to 400 m. The literature on this topic is not homogeneous, with studies reporting a mean range of *Ae. albopictus* male dispersal not exceeding 100 m [38,39] and others highlighting a higher range of spread [30,32], but, generally, most of the males remain within 200 m of the release spot.

Regarding the field longevity of AR*w*P males, results were noticeable since the number of incompatible males did not significantly decrease between 3 and 11 days after the release, and males were found alive up to 18 days after the release (Figure S7). This observation, even if merely qualitative, agrees with the previous literature estimating an *Ae. albopictus* average life expectancy of 5–10 days, depending on the period of the year [38], while other studies evidenced a shorter lifespan [39]. A good longevity means multiple chances for a male to inseminate virgin wild females, resulting in a cumulative impact on the mean egg fertility of the target population. Although these data do not allow for definitive conclusions regarding the dispersal capacity or the fitness of AR*w*P males, they nonetheless offer valuable insights into their dispersal behavior and the effective range of induction of sterility.

Provided information may serve as an indication for the setup of effective and sustainable IIT protocols to design a release scheme capable of optimizing the number of release spots per unit area, the number of incompatible males to be released per release spot, and the time interval between subsequent releases. Setting a 200 m distance between release spots and 1-week intervals between releases could be a good option to be applied under urban and peri-urban settings to try to combine effectiveness and sustainability. Nevertheless, it is important to emphasize that reported observations are context- and time-dependent, being influenced by the ratio between released and wild males, by the specific environmental characteristics of the study site, and by the seasonality. Indeed, urban mosquito populations are highly heterogeneous in terms of density, due to the varying microclimatic conditions and habitat fragmentation [40,41], and these factors directly affect male dispersal, survival, and mating success [32]. Also, considering the seasonal dynamics of the local climate during IIT programs is fundamental because temperature and humidity can significantly affect *Ae. albopictus* longevity [42,43]. In this regard, the warm and dry conditions characterizing most of the Mediterranean climate summer are expected to significantly affect *Ae. albopictus* male fitness regardless of the *Wolbachia* infection type, but setting the incompatible male release frequency to twice a week, instead of once a week, could be a better option to ensure constant pressure on the wild population. A comparison between the male mating competitiveness of incompatible and wild males under stressing environmental conditions can be the object of further research to further improve IIT protocols to be implemented in Europe.

Additional studies could investigate the possibility of integrating IIT with other compatible control measures [44,45] to set up effective and sustainable Integrated Vector Management programs suitable for the specific conditions of urban areas in Mediterranean Europe.

## 5. Conclusions

This study demonstrates that non-inundative, single-spot releases of incompatible AR*w*P males can effectively reduce the egg fertility of wild *Ae. albopictus* populations over a large area, with the greatest impact observed within 100 m of the release spot but with a detectable sterilizing gradient extending up to 500 m. Recapture data confirm that although some AR*w*P males can disperse several hundred meters, their density, survival, and sterilizing impact are highest near the release spot. The experimental framework adopted here, which exploits the molecular traceability of *Ae. albopictus* AR*w*P males, represents a valuable tool for studying male dispersal and the spatial pattern of induced egg sterility. This approach is certainly easier and safer for male fitness compared to MRR methods and may represent a further addition to the array of methods already designed to overcome MRR limitations [46,47]. The findings can contribute to optimizing release strategies by ensuring effective coverage and maximizing the operational efficiency of IIT, especially in scaled-up applications. While climatic factors and release timing may have influenced the observed population dynamics, further research is needed to disentangle these effects and to evaluate the potential long-term or cumulative impacts of repeated AR*w*P male releases on wild *Ae. albopictus* populations.

## Figures and Tables

**Table 1:** Observed AR*w*P:wild *Ae. albopictus* male ratio as a function of distance and time after the last incompatible male release occurred in June 2023 at ENEA-Casaccia Research Center (Rome, Italy). The actual count of, respectively, AR*w*P and wild males is reported in brackets.

			AR*w*P:wild Male Ratio as a Function of Distance and Time				
Date	Released AR*w*P Males	Days After the Last Release	0–100 m	100–200 m	200–300 m	300–400 m	400–500 m
22/06/23	9000	-	-	-	-	-	-
28/06/23	15,000	-	-	-	-	-	-
01/07/23	-	3	1.58 (19:12)	0.77 (7:9)	0.09 (1:11)	0.00 (0:31)	0.00 (0:14)
08/07/23	-	11	1.73 (26:13)	0.15 (2:13)	0.35 (6:17)	0.13 (3:22)	0.00 (0:17)
15/07/23	-	18	0.11 (2:18)	0.07 (2:26)	0.14 (1:7)	0.00 (0:16)	0.00 (0:26)
22/07/23	-	22	0.00 (0:14)	0.00 (0:17)	0.00 (0:14)	0.00 (0:11)	0.00 (0:10)

## Data Availability

The original contributions presented in this study are included in the article/Supplementary Materials. Further inquiries can be directed to the corresponding authors.

## Supplementary Materials

The following supporting information can be downloaded at https://www.mdpi.com/article/10.3390/tropicalmed11020045/s1. Table S1: Number of ovitraps located at different distances from the AR*w*P release spot; Figure S1: Temporal line relative to the 2022 trials; Figure S2: Temporal line relative to the 2023 trials; Figure S3: Temporal line relative to the collection of single *Ae. albopictus* females conducted in 2023; Figure S4: Pearson residuals versus fitted values (A,C,E) and normal quantile–quantile (QQ) plots (B,D,F) for GLMs; Table S2: Comparison between the hatching rates of *Ae. albopictus* eggs collected by ovitraps (*N* = 58) in a 2-week interval before AR*w*P male releases (pre-treatment phase) and after AR*w*P male releases (post-treatment phase) in 2022 (GLM-1) and 2023 (GLM-2); Table S3: Estimated mean reduction (%) of the hatching rates of *Ae. albopictus* eggs collected by ovitraps (*N* = 58) comparing 2-week intervals before AR*w*P male releases (pre-treatment phase) and after AR*w*P male releases (post-treatment phase) in 2022 (GLM-1) and 2023 (GLM-2); Table S4: Number of single wild *Ae. albopictus* females and produced eggs (in brackets) analyzed before and after the last non-inundative release of AR*w*P males (28th June) in the study area (ENEA-Casaccia) in summer 2023 at different distances from the AR*w*P release spot; Table S5: Comparison of the hatching rates of the eggs laid by single wild *Ae. albopictus* females collected before (pre-treatment phase) and after (post-treatment phase) the last AR*w*P male release occurred in 2023 (GLM-3); Table S6: Mean difference (%) between the hatching rates of eggs laid by single wild *Ae. albopictus* females collected before (pre-treatment phase) and after (post-treatment phase) the last AR*w*P male release occurred in 2023 (GLM-3), at different distances (m) from the AR*w*P male release spot. CI = 95% of confidence interval; Figure S5: Weekly average temperature (C°) and Precipitation (mm) during the trials in 2022 and 2023; Figure S6: Average number of eggs (black) weekly collected by ovitraps (*N* = 58) in all the study area and proportion of the eggs that hatched (red) with 95% confidence interval in 2022 (left) and 2023 (right); Table S7: Summary of the GLM-4 model, *** = significant *p* value < 0.001.; Figure S7: Estimates of mean proportion of hatching eggs out of the eggs laid by single wild *Ae. albopictus* females collected before (pre-treatment phase, in blue) and after (post-treatment phase, in black, green and red) the *Ae. albopictus* AR*w*P male releases of 2023, as a function of distance from the release spot, obtained by GLM-5.
