# Supplementary material for: Spatial Dynamics and Sterilization Range of Incompatible Aedes albopictus Males: Advancing Toward an Optimized IIT Approach
Source: Trop Med Infect Dis. 2026 Feb 6;11(2):45. doi: 10.3390/tropicalmed11020045 (PMC12944944; doi:10.3390/tropicalmed11020045)
Supplement: Supplementary file 1 [file tropicalmed-11-00045-s001.zip › tropicalmed-4100915-supplementary.pdf]

Supplementary Materials

Spatial dynamics and effective sterilization range of incompatible *Aedes albopictus* males: advancing toward an optimized IIT approach

Elena Lampazzi [1], Chiara Virgillito [2], Beniamino Caputo [2], Giulia Lombardi [1], Greta Santarelli [2], Riccardo Moretti [1*], and Maurizio Calvitti [1*]

[1] Italian National Agency for New Technologies, Energy and Sustainable Economic Development (ENEA), Rome, Italy; elena.lampazzi@enea.it (EL); riccardo.moretti@enea.it (RM); giulia.lombardi.1993@gmail.com (GL); maurizio.calvitti@enea.it (MC)

[2] Department of Public Health and Infectious Diseases, Sapienza Università di Roma, Roma, Italy; chiara.virgillito@uniroma1.it; beniamino.caputo@uniroma1.it; greta.santarelli@uniroma1.it

* Correspondence: riccardo.morei@e-mail.com, tel.: +39 0630486584; maurizio.calvitti@enea.it, tel.: +39 0630484597.

Table S1: Number of ovitraps located at different distance from the AR*w*P release spot (N=58)

	N ovitraps	
Distance from the AR*w*P release spot (m)	**2022**	**2023**
0-100	16	16
100-200	12	12
200-300	6	6
300-400	7	7
400-500	2	3
500-600	2	2
600-700	3	3
700-800	7	7
800-900	3	3

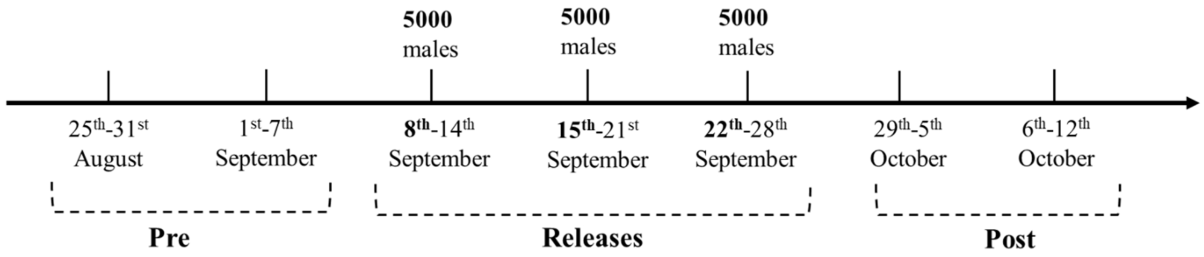

Figure S1: Temporal line relative to the 2022 trials describing pre- and post- treatment phases (respectively, Pre and Post) and the treatment phase (Releases) consisting in the release of *Ae. albopictus* AR*w*P males. The days of the releases are indicated in bold.

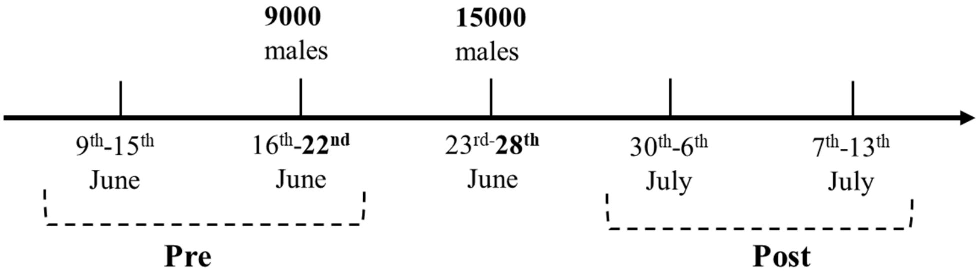

Figure S2: Temporal line relative to the 2023 trials describing pre- and post- treatment phases (respectively, Pre and Post) and the treatment phase (Releases) consisting in the release of *Ae. albopictus* AR*w*P males. The days of the releases are indicated in bold.

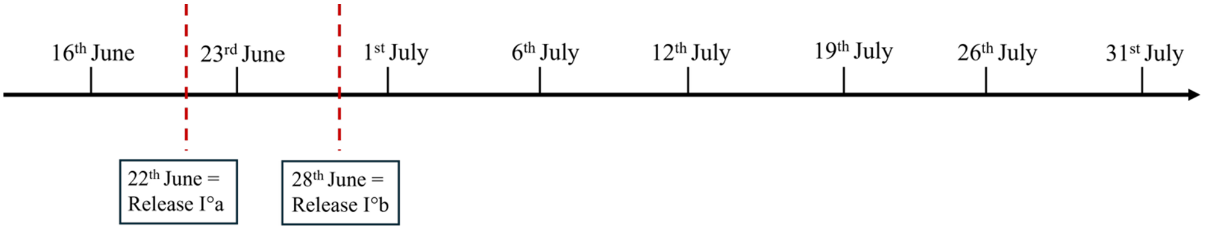

Figure S3: Temporal line relative to the collection of single *Ae. albopictus* females conducted in 2023. Red lines = date of the two AR*w*P male releases occurred in 2023. Release I°a = 9,000 AR*w*P males; Release I°b=15,000 AR*w*P males

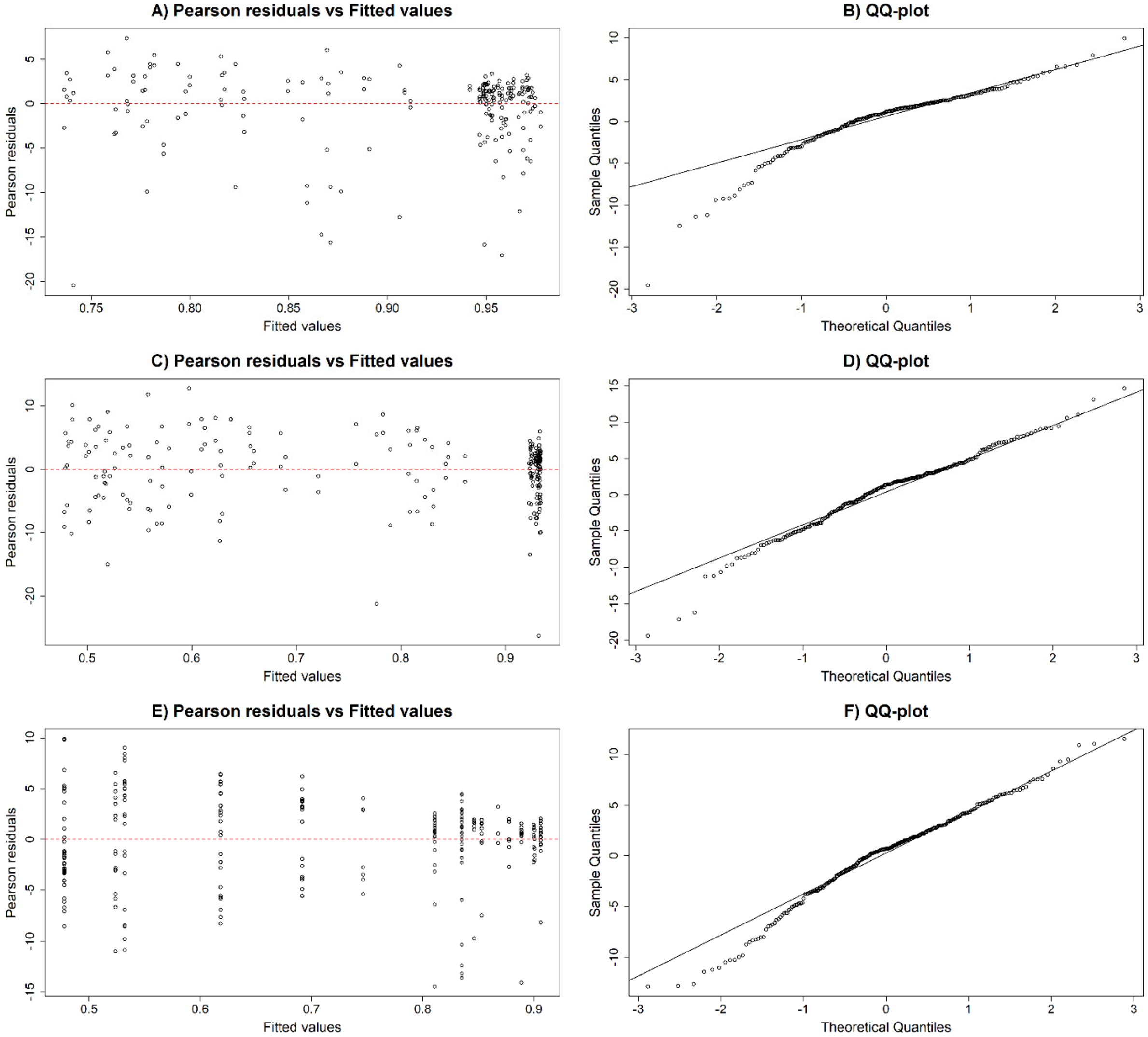

Figure S4: Pearson residuals versus fitted values (A, C, E) and normal quantile–quantile (QQ) plots (B, D, F) for GLMs. Top row: GLM-1 (A–B); middle row: GLM-2 (C–D); bottom row: GLM-3 (E–F). Dashed red horizontal line (left panels) = zero residuals. Diagonal reference line (right panels) = theoretical quantiles under normality.

Table S2: Comparison between the hatching rates of *Ae. albopictus* eggs collected by ovitraps (N=58) in a 2-weeks interval before AR*w*P male releases (pre-treatment phase) and after AR*w*P male releases (post-treatment phase) in 2022 (GLM-1) and 2023 (GLM-2). Baseline on the intercept= pre. In 2022, AR*w*P males releases were carried out on September 8th, 15th and 22nd (N tot=15,000); In 2023, AR*w*P males releases were carried out on June 22nd and 28th (N tot=24,000).

Year of AR*w*P releases	Coefficient	Estimate	Std. Error	z value	Pr (>|z|)
2022	Intercept	2,646	0,053	5,22	<2e-16***
	post	-2,778	0,0567	-48,95	<2e-16***
	distance	-0,00019	0,00012	-1,543	0,123
	Post*distance	0,0023	0,00014	17,32	<2e-16***
2023	Intercept	2,859	0,08	34,35	<2e-16***
	post	-1,89	0,092	-20,488	<2e-16***
	distance	0,0009	0,0002	4,423	9,72e-06***
	Post*distance	0,0022	0,00024	9,323	<2e-16***

Table S3: Estimated mean reduction (%) of the hatching rates of *Ae. albopictus* eggs collected by ovitraps (N = 58) comparing 2-weeks intervals before AR*w*P male releases (pre-treatment phase) and after AR*w*P male releases (post-treatment phase) in 2022 (GLM-1) and 2023 (GLM-2) with 95% CI as function of distance from the release spot in 2022 (GLM-1) and 2023 (GLM-2).

	2022	2023
Class of distance from the release spot (m)	Egg hatching reduction (%) comparing pre- and post-treatment phases (95%CI)	Egg hatching reduction (%) comparing pre- and post-treatment phases (95%CI)
0-100	46.5 (45.3, 47.6)	19.9 (18.8, 21)
100-200	41 (39.8, 42.1)	15.1 (14.2, 16)
200-300	35.3 (34.2, 36.4)	11 (10.2, 11.7)
300-400	29.9 (28.8, 30.9)	7.7 (7.1, 8.3)
400-500	24.5 (23.5, 25.6)	5.1 (4.6, 5.5)
500-600	19.6 (18.7, 20.5)	3.1 (2.8, 3.4)
600-00	15.4 (14.6, 16.2)	1.7 (1.5, 1.9)
700-800	11.6 (10.9, 12.3)	0.7 (0.6, 0.9)
(800,908]	8.1 (7.5, 8.8)	0

Table S4: Number of single wild *Ae. albopictus* females and produced eggs (in brackets) analysed before and after the last non-inundative release of AR*w*P males (28th June) in the study area (ENEA-Casaccia) in summer 2023 at different distances from the AR*w*P release spot.

	June	July		
Distance from the AR*w*P release spot (m)	16th	1st (2 days after release)	6th (7 days after release)	12th (14 days after release)
0-100	15 (N=517)	14 (N=670)	13 (N=577)	14 (N=606)
100-200	13 (N=480)	12 (N=575)	13 (N=548)	15 (N=872)
200-300	10 (N=400)	10 (N=364)	9 (N=424)	9 (N=302)
300-400	6 (N=169)	6 (N=180)	6 (N=285)	7 (N=224)
400-500	3 (N=126)	3 (N=151)	2 (N=79)	3 (N=136)
600-700	18 (N=474)	18 (N=759)	19 (N=867)	17 (N=582)

Table S5: Comparison of the hatching rates of the eggs laid by single wild *Ae albopictus* females collected before (pre-treatment phase) and after (post-treatment phase) the last AR*w*P male release occurred in 2023 (GLM-3). Baseline on the intercept= pre.

Coefficient	Estimate	Std. Error	z value	Pr (>|z|)
Intercept	2,33	0,12	19,45	<2e-16***
post	-2.61	0.13	-20.75	<2e-16***
Distance	-0,0009	0,00029	-3,085	0,002**
post*distance	0,004	0,0004	11.71	<2e-16***

Table S6: Mean difference (%) between the hatching rates of eggs laid by single wild *Ae albopictus* females collected before (pre-treatment phase) and after (post-treatment phase) the last AR*w*P male release occurred in 2023 (GLM-3), at different distance (m) from the AR*w*P males release spot. CI=95% of confidence interval.

Distance from AR*w*P release spot (m)	Reduction (%) of the hatching rate	95% CI
70	47.3	(44.9,49.6)
138	41.8	(39.7, 43.9)
150	40.9	(38.8, 42.9)
280	30.5	(28.7, 32.2)
400	21.2	(19.3, 23.0)
500	13.9	(11.8, 16.2)
638	4.9	(1.9, 7.9)
700	1.3	(-2.2, 4.7)

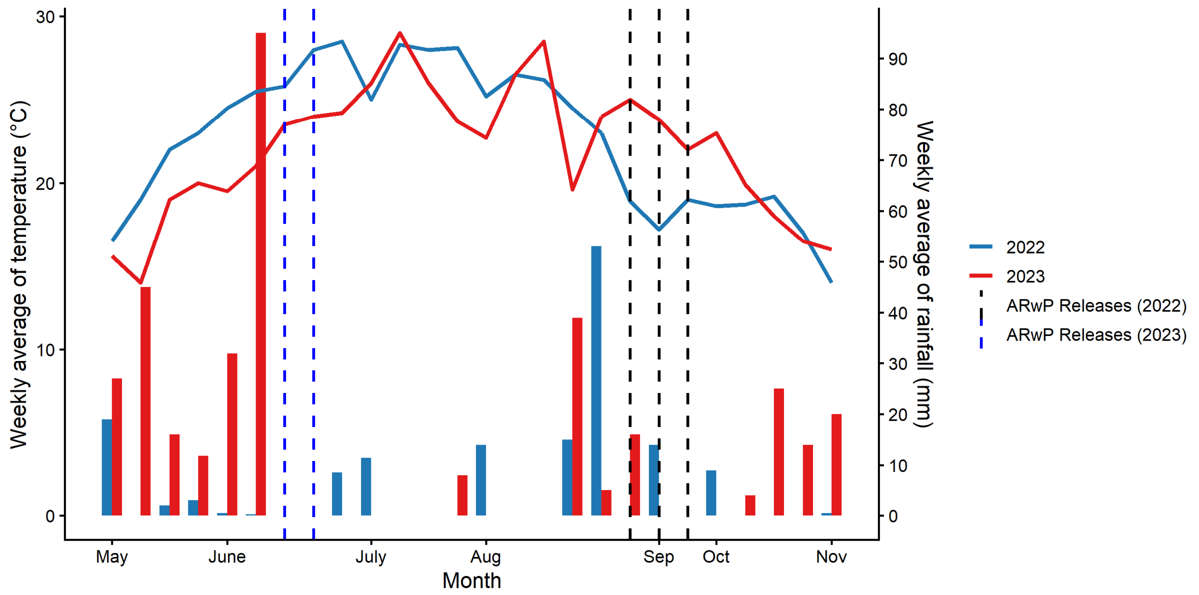

Figure S5: Weekly average temperature (C°) (line, Right y-axis) and Precipitation (mm) (bars, Left y-axis) during the trials in 2022 (blue) and 2023 (red). Black dashed lines = AR*w*P males releases in 2022 (5,000 ARwP males for each release). Blue dashed lines = AR*w*P males releases in 2023 (9,000 and 15,000 males)

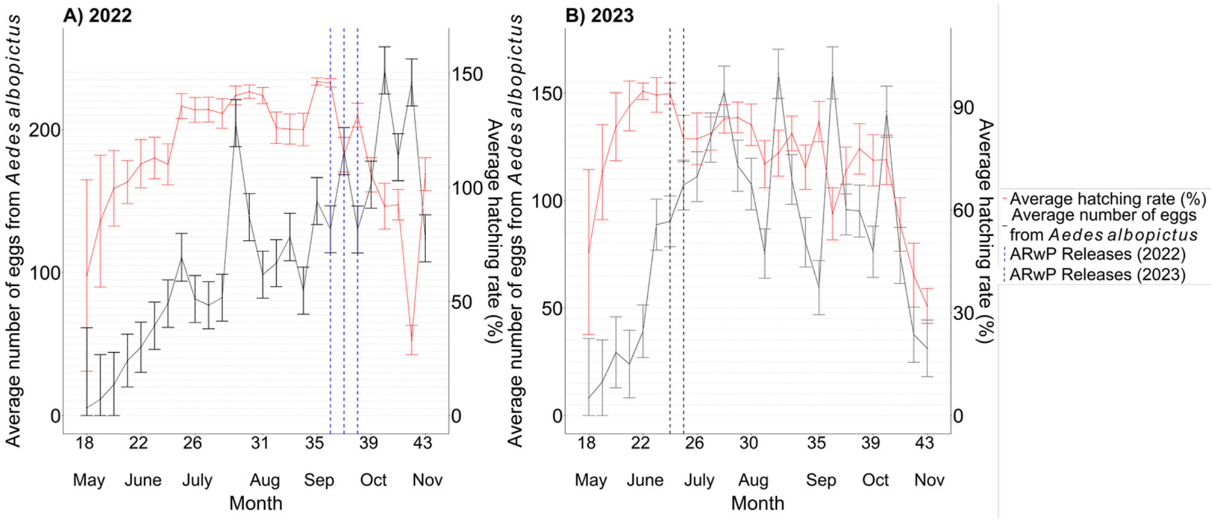

Figure S6: Average number of eggs (black) weekly collected by ovitraps (N=58) in all the study area and proportion of the eggs that hatched (red) with 95% confidence interval in 2022 (A) and 2023 (B). Dark Blue dashed lines = AR*w*P males releases in 2022 (5,000 ARwP males for each release). Blue, Cyran dashed lines = AR*w*P males releases in 2023 (9,000 and 15,000 males)

Table S7: Summary of the GLM-4 model. The GLM-4 model was implemented to evaluate how the reduction of egg-hatching rates changed every 100 m from the release spot between pre- and post-treatment phases periods in the two years of study. The response variable was defined as follows: for each 100-m distance class, the reduction estimates obtained from GLM-1 and GLM-2 (Table S3) were subtracted from the reduction estimate of the next distance class (e.g., the 46% reduction at 0–100 m was subtracted from the 41% reduction at 100–200 m), yielding the change in reduction between consecutive 100-m classes. The response variable was modelled using a Gamma distribution. Year was included as a fixed effect, with 2022 used as the baseline. ***= significant p value < 0.001.

Coefficient	Estimate	Std. Error	z value	Pr (>|z|)
Intercept	1,56	0,15	10,07	<2e-16***
year:2023	-0,65	0.22	-2,9	0,009***

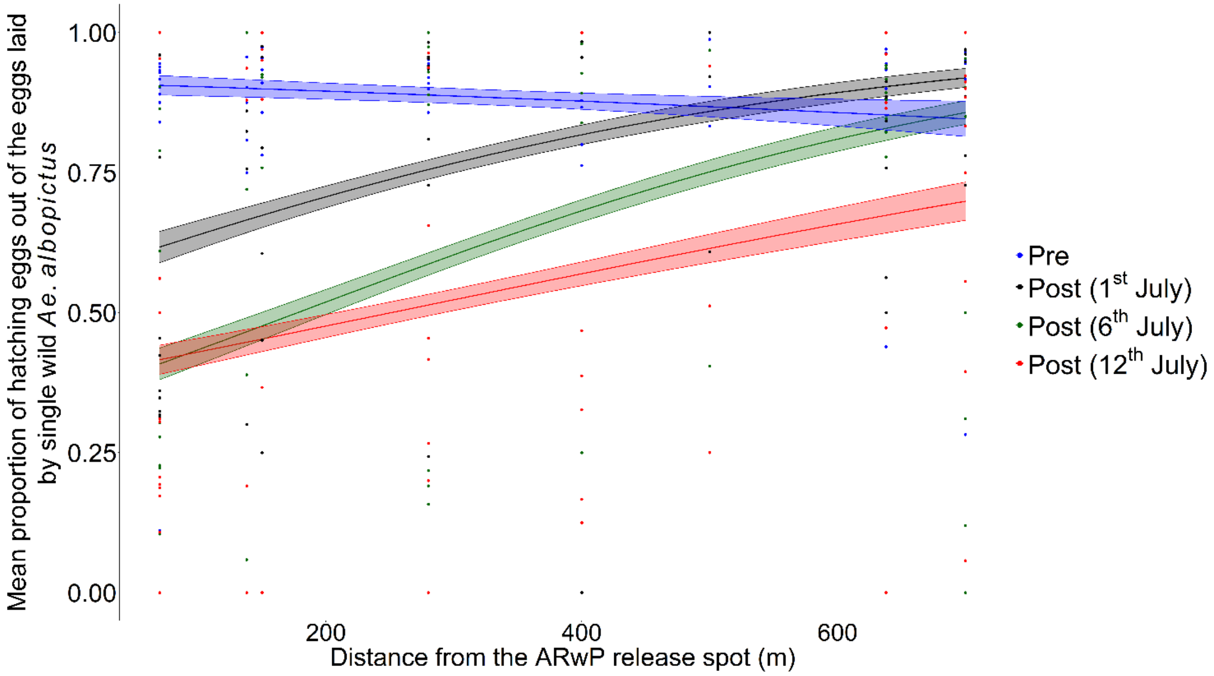

Figure S7: Estimates of mean proportion of hatching eggs out of the eggs laid by single wild *Ae. albopictus* females collected before (pre-treatment phase, in blue) and after (post-treatment phase, in black, green and red) the *Ae. albopictus* AR*w*P male releases of 2023, as a function of distance from the release spot, obtained by GLM-5. The GLM-5 had the same statistical structure of GLM-3 however it included the three dates of female collection as separate levels to estimate the effect of the *Wolbachia*-induced sterilization as a function of time. Dots = observed proportion of hatching eggs. Pre: June 16th, in blue; post: July 1st in black, July 6th in green, July 12th in red.
